# Antioxidant activity of pomegranate juice reduces acute lung injury secondary to hyperoxia in an animal model

**DOI:** 10.1186/1756-0500-7-664

**Published:** 2014-09-21

**Authors:** Ahmad Husari, Aline Khayat, Hala Bitar, Yasmine Hashem, Alain Rizkallah, Ghazi Zaatari, Marwan El Sabban

**Affiliations:** Division of Pulmonary and Critical Care Medicine, Department of Internal Medicine, American University of Beirut, P.O. Box 11-236, Riad El Solh, Beirut, 1107 2020 Lebanon; Department of Pathology & Laboratory Medicine, American University of Beirut, Beirut, Lebanon; Department of Biochemistry, American University of Beirut, Beirut, Lebanon

**Keywords:** Reactive oxygen species, Antioxidants, Acute lung injury, Hyperoxia, Inflammatory mediators

## Abstract

**Background:**

Hyperoxia triggers the release of toxic reactive oxygen species (ROS). Pomegranate Juice (PJ) is a rich source of potent antioxidants. We assessed the effects of PJ supplementation on Acute Lung Injury (ALI) in adult rats exposed to hyperoxia for 5 days.

**Methods:**

Adult rats were divided into four different groups: control, hyperoxia, hyperoxia + PJ and PJ. Animals were placed in chambers containing either room air or oxygen above 95% for a total of 5 days. Two different PJ concentrations were utilized and the control group received placebo water. Animals were euthanized and their lungs were excised. Assessment of lung injury was accomplished by: a) wet to dry ratio (W/D) method, b) measurement of albumin concentration in the bronchoalveolar lavage fluid (BALF), c) oxidative stress, d) histological evaluation of the lung e) apoptosis and f) transcriptional expression levels of the inflammatory mediators IL-1β, IL-6 and TNF-alpha.

**Results:**

An increase in the W/D and albumin leak was noted in Hyperoxia (p < 0.05). Those findings were attenuated by the higher dose of PJ supplementation. Hyperoxia increased ROS production. Again PJ significantly reduced oxidative stress. Lung sections showed significant reduction in inflammation, edema, and infiltrating neutrophils in Hyperoxia + 80 μmol/kg when compared with Hyperoxia. TUNEL demonstrated significant apoptosis in the Hyperoxia, which was diminished in the Hyperoxia + 80 μmol/kg. Furthermore, increase in IL-1β and IL-6 was noted in Hyperoxia. Again, 80 μmol/kg of PJ significantly reduced the expression of inflammatory mediators.

**Conclusion:**

In this animal model, PJ supplementation attenuated ALI associated with hyperoxia.

## Background

Oxygen is frequently administered to patients with hypoxia or in respiratory distress. Hyperoxia, on the other hand, can also be detrimental and may worsen acute lung injury (ALI) in patients with acute respiratory distress syndrome and in infants with bronchopulmonary dysplasia [1, 2]. The damaging effects of hyperoxia are associated with excessive generation of hydroxyl radicals, superoxide and hydrogen peroxide molecules [2, 3]. These reactive oxygen species (ROS) may oxidize nucleic acids, membrane lipids and proteins leading to cellular damage and death [4, 5]. Further recruitment of neutrophils and infiltration of lung parenchyma is noted with prolonged hyperoxia that will persist even after the termination of oxygen exposure [6]. Cellular defenses against increased oxidative stress such as antioxidant enzymes and non-protein thiols become depleted by the excess production of ROS instigating cellular injury and death [7].

To prevent oxidative stress/imbalance noted in hyperoxia, antioxidant supplementation in animal models and in humans was explored with mixed results [8, 9]. Crapo et al. tested a catalytic antioxidant metalloporphyrin (AEOL 10113) on fetal baboons receiving 100% oxygen. AEOL 10113 supplementation was associated with a significant reduction in the number of mast cells and eosinophils infiltrating the lungs and a reduction in alveolar septa thickness noted with hyperoxia [10]. Transgenic neonatal mice expressing extra copy of extracellular superoxide dismutase (EC-SOD) had a significant reduction in ROS and improved angiogenesis in neonatal mice lung following exposure to hyperoxia, while EC-SOD knocked out mice had reduced neovascularization, increased endothelial dysfunction and worsening lung injury when compared to controls [11, 12]. The promising data from animal studies have not translated to significant results in human trails [13–15].

*Punica granatum* L. (Punicaceae), referred to as pomegranate, is commonly consumed as pomegranate juice (PJ). It is well known that PJ possesses exceptional antioxidant, antiatherogenic, antiproliferative and anti-inflammatory effects [16]. PJ contains bioactive compounds that are powerful antioxidants, particularly polyphenols. Punicalagins α and β monomers are predominant ellagitannins responsible for the antioxidant properties of PJ. In addition, the combination of punicalagins α and β with other phytochemicals such as ellagic acid and its glycosylated derivatives synergistically enhances the superior antioxidant properties of PJ [17]. When compared to other polyphenol rich juices or beverages, PJ has the highest antioxidant properties [18].

In this study, we examined the role of antioxidants, represented by PJ, in attenuating the pathological and inflammatory parameters of ALI in an animal model exposed to hyperoxia.

## Methods

The study was reviewed and approved by the university research board and the institutional animal care and use committee of the American University Of Beirut. Two month old Sprague–Dawley rats were distributed to litters of 12 of equal body weight and nested on softwood shavings in plexiglas exposure chambers. Food and water were made available *ad libitum* and lighting was provided on a 12-hr light/dark cycle. Room and chambers temperature were maintained at 22-24°C. The chambers were equipped with flow-through systems for controlling the delivery of either medical oxygen or room air. In chambers receiving oxygen, medical oxygen concentration was continuously monitored using an oxygen monitor (Ohmeda 5120, Douisville, CO) and maintained at 95 ± 2%. Carbon dioxide (CO_2_) levels were maintained at 0.3% by placing soda lime inside the chamber to absorb CO_2_. Air within the chamber was recycled after being passed through anhydrous calcium sulfate and silica gel mixture to remove ammonia and moisture. Relative humidity was maintained between 50% and 80%. Animals were divided into four different groups: control, hyperoxia, hyperoxia + PJ and PJ. The last two groups were repeated for two different concentrations of PJ (PJ40 and PJ80).

PJ concentrate (Wonderful variety, POM Wonderful, Los Angeles) was utilized in this study. Compared with other commercially available PJ, this product is well studied and its antioxidant polyphenols are characterized and verified [19]. The preparation of PJ is described by the manufacturer and the dosage was extrapolated from previous studies [20]. Concentrated PJ was diluted in water and administered in two different concentrations: a) 40 μmol of polyphenols from PJ/kg of body weight (PJ40) and b) 80 μmol of polyphenols from PJ/kg of body weight (PJ80). The appropriate dose of PJ was prepared daily and mixed with the drinking water. Daily fluid intake was observed for all animals. The other groups received placebo free water. PJ or water supplementation was initiated two weeks prior to hyperoxia and maintained throughout the experiment. Animals were then exposed to either room air or hyperoxia for a total of five days.

Animals were anesthetized with xylazine and ketamine. The trachea was cannulated with polyethylene tubing. The abdomen was opened and animals were exsanguinated by severing the aorta. The diaphragm was dissected to allow free lung expansion. Bronchoalveolar lavage fluid (BALF) samples were obtained by clipping the left lower lobes and lavaging the remaining lung lobes by slowly instilling 1.5 ml of warm PBS (Ca^++^ and Mg^++^ free, 37°C) and then gently aspirating. This cycle was repeated three times. The clipped left lower lobes were excised for pulmonary water content evaluation. The left upper lobes were fixed in formalin for pathology examination, ROS and TUNEL assay. The remaining right lobes were individually frozen in liquid nitrogen for RNA extraction and RT-PCR.

### Assessment of ALI

#### Wet-to-dry lung weight

Left lower lobes were excised, weighed and then placed in the oven at 90°C for 48 hours. The dry weight was obtained and the W/D was calculated.

#### Bronchoalveolar lavage (BAL): cell count and albumin level

BALF was centrifuged (4020 rpm for 5mins at 25°C) onditions and the supernatant was stored at -80°C. The BAL cell pellet was resuspended in 1 ml of 0.9% NaCl, and the total number of cells was determined by counting cells on a hemocytometer. Differential cell counts (400 cells per slide) were performed on cytospin-prepared slides (Thermo Shandon, Pittsburgh, PA) stained with Diff-Quik (Dade Behring, Newark, DE). Trypan blue was utilized to exclude any sample that has less than 90% viability of cells.

The supernatant BALF was utilized for albumin measurement. The concentration of albumin was determined by an immune-turbidimetric assay using polyclonal anti-albumin antibodies (Roche Applied Science, Indianapolis, IN, USA). Agglutination, caused by antigen/antibody complexes, was measured utilizing a Hitachi 912 Autoanalyser (Roche Diagnostics, Basel, Switzerland).

#### Detection of superoxide production

Dihydroethydine (DHE) was used to localize superoxide production. Briefly, lung tissues (5 μm thick) were obtained from (n = 4) control, hyperoxia, hyperoxia + PJ and PJ. Lung sections were incubated with DHE (10 μmol/L dissolved in DMSO, incubated in light-protected humidified chamber at 37°C for 15 min). Sections were not washed and directly visualized by fluorescence microscopy (Zeiss 710 laser scanning confocal microscope).

#### Measurement of inflammatory mediators IL-1β, IL-6, and TNF-alpha

Inflammatory mediators’ transcriptional levels were assessed using reverse transcriptase- polymerase chain reaction (RT-PCR) method. Total RNA was isolated from the lung homogenates using the TRIZOL method (Invitrogen, Carlsbad, CA, USA). Briefly, 1 ml of TRIZOL reagent was used per 50 - 100 mg of tissue sample followed by chloroform extraction. RNA samples were precipitated and stored at -80°C. RNA was quantified using a 260/280 nm absorbance ratio method. Total RNA (1 μg) was reverse-transcribed into first strand cDNA using a special kit (Ready-to-go You-prime-Strand-Beads, GE healthcare, UK). Real time-PCR was performed using the iCycler (Bio-Rad laboratories, Hercules, CA, USA) with SYBR Green. Specific primers (Tib-Molbiol, Berlin, Germany) were utilized to assess the expression of inflammatory mediators in these tissues:

- (*IL-1β*: Fw CACCTCTCAAGCAGAGCACAG, Rw GGGTTCCATGGTGAAGTCAAC;
- *IL-6:* Fw TCCTACCCCAACTTCCAATGCTC, Rw TTGGATGGTCTTGGTCCTTAGCC;
- *TNF-α*: Fw AATGGGCTCCCTCTCATCAGTTC, Rw TCTGCTTGGTGGTTTGCTACGAC;

*GAPDH*: Fw GTATTGGGCGCCTGGTCACC, Rw CGCTCCTGGAAGATGGTGATGG).

PCR products and their corresponding melting temperatures were analyzed using the iQ5 Optical System Software (Bio-Rad laboratories). Corrections for loading were achieved by subtraction of local background and normalization against the cDNA levels of the GAPDH housekeeping gene.

#### Assessment of apoptosis

The Terminal deoxynucleotidyl transferase-mediated dUTP Nick-End Labeling (TUNEL) assay was used to monitor the extent of DNA fragmentation as a measure of apoptosis in paraffin-embedded sections. The assay was performed according to the recommendations of the manufacturer (Boehringer, Mannheim, Germany). Paraffin sections of 4 μm thick were used and Fluorescein-conjugated dUTP incorporated in nucleotide polymers were scanned for signal using 488 nm excitation line from the Zeiss LSM 410 laser scanning confocal microscope. Positive and negative controls were used to verify the specificity of the TUNEL assay. Positive controls were treated with DNase I (Sigma Chemical Co. Saint-Louis, MI, USA) to enzymatically induce DNA fragmentation. TUNEL-positive nuclei were distinguished from the TUNEL-negative nuclei by counter staining with Hoechst 33258 (Molecular Probes, Eugene, OR).

#### Pathologic evaluation of ALI

The upper lobe of the right lung was harvested and immersed in 4% formaldehyde solution. The tissue was then paraffin embedded, serially sectioned, and stained with hematoxylin and eosin (H&E). Pulmonary morphologic characteristics were evaluated as described before [6]. Briefly, a pathologist, blinded to different animal groups, evaluated the histopathologic findings under light microscopy and determined the presence of lung injury based on the following histological features: septal edema, congestion, degree of inflammatory cell infiltration, and alveolar edema.

#### Statistical assessments

Differences were considered significant for p < 0.05 in comparing two measurements. Comparison among groups was evaluated by analysis of variance (ANOVA). The Kruskall-Wallis test was used to compare apoptotic index scores.

## Results

There was no significant difference in the daily fluid intake of the different animal groups. An increase in mortality in the hyperoxia only group (25%) and at the end of the experiment, the remaining animals appeared ill and lost weight.

### Body weight, lung weight and W/D ratio

**Figure 1:**
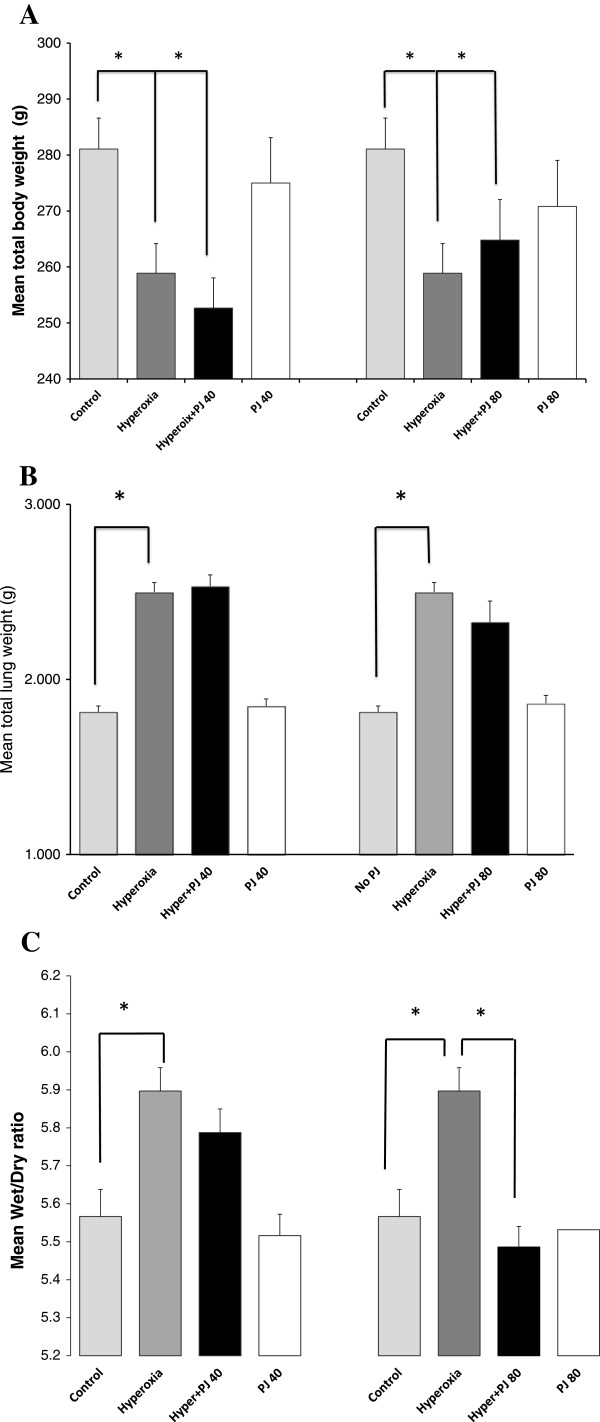
**Mean total body weight (A), total lung weight (B), and W/D (C) of control, hyperoxia, hyperoxia + PJ40 and hyperoxia + PJ80.** Error bars represent SE. Sample size is eight per group. Asterisks indicate statistically significant associations (P < 0.05).

There was a statistically significant decrease in total body weight (TBW) of animals exposed to hyperoxia when compared to the control group (P = 0.009). Supplementation with PJ (PJ40 and PJ80) failed to reverse the loss of weight associated with hyperoxia (Figure 1A). Likewise, animals exposed to hyperoxia demonstrated a significant increase in mean total lung weight when compared to the control group (P = 4.9 × 10^-11^) (Figure 1B). There was no difference in mean W/D between the control group and the PJ40 or PJ80 groups (P = 0.6 and P = 0.8 respectively). There was, however, a statistically significant increase in mean W/D noted in the hyperoxia group when compared to the control group (P = 0.00004). PJ supplementation did attenuate the increase in W/D in the hyperoxia + PJ80 when compared to hyperoxia (P = 0.0003) (Figure 1C).

### Bronchoalveolar lavage: cell count and albumin level

A statistically significant increase in the number of RBC and WBC was observed in hyperoxia when compared to controls (P = 0.0001 and P = 0.03 respectively). The supplementation of PJ to animals exposed to hyperoxia demonstrated a trend in reducing the number of RBC and WBC in BALF. This trend, however, didn’t reach a statistical significance for both PJ doses utilized in this study. Upon examination of BALF slides, we have observed an increase in cellularity and neutrophil abundance in hyperoxic groups. This was decreased but not completely recovered upon administration of POM in a dose dependendant manner.

**Figure 2:**
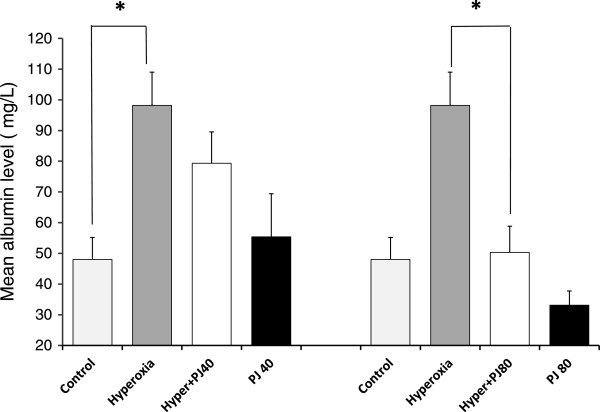
**Mean albumin level in the BALF of control, hyperoxia, hyperoxia + PJ40 and hyperoxia + PJ80.** Error bars represent SE. Sample size is eight per group. Asterisks indicate statistically significant associations (P < 0.05).

Albumin leak in BALF increased significantly with hyperoxia (P = 0.0005) when compared to the control group. When compared to hyperoxia, albumin leak was significantly attenuated with the higher concentration of PJ only (Hyperoxia + PJ80) (P = 0.004; Figure 2).

### ROS generation

**Figure 3:**
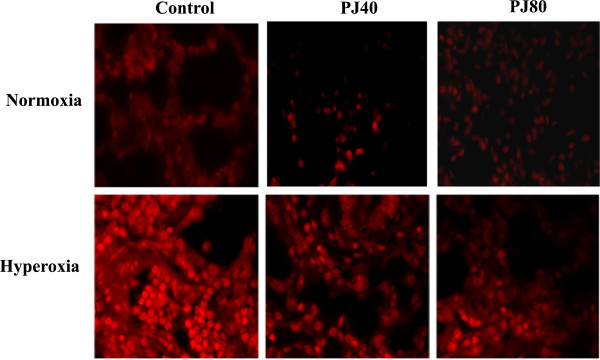
**ROS detection in lung tissues.** 5 μm thickness slides were incubated with Dihydroethidium (DHE) as described by the manufacturer. Fluorescent images of ethidium-stained tissue were analyzed using Zeiss 710 laser scanning confocal microscope. ROS levels were induced in hyperoxia treated animals. Levels of ROS were attenuated in lung tissues of PJ treated animals.

Control and PJ groups demonstrated low levels of the oxidative stress. In response to hyperoxia, there was an increase in fluorescence indicating that hyperoxia created increased ROS production. In animals pre-treated with PJ and exposed to hyperoxia, the amount of oxidative stress was significantly lower than animals exposed to hyperoxia and not different from the control groups. This indicated that PJ supplementation preserved oxidative balance and reduced the oxidative stress associated with hyperoxia (Figure 3).

### Inflammatory mediators

**Figure 4:**
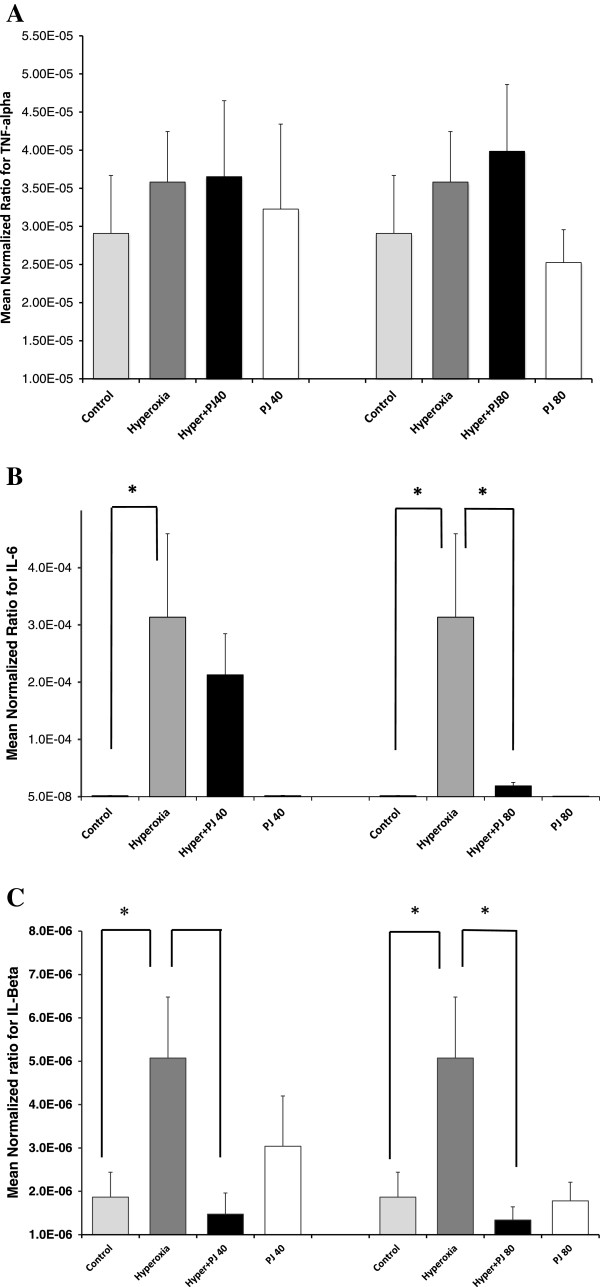
**Transcriptional expression TNF-α (A), IL-6 (B) and IL-1β (C).** Expression of inflammatory mediators was evaluated by real-time PCR in animals of control, hyperoxia, hyperoxia + PJ40 and hyperoxia + PJ80. Sample size is eight per group. Bars represent SE. Asterisks indicate statistically significant associations (P < 0.05).

There was no difference in TNF-α expression between control and PJ groups. In addition and after 5 days of hyperoxia exposure, there was no significant increase in TNF-α expression when compared to the control group (Figure 4A). Likewise, there was no difference in IL-1β expression between controls and PJ groups. The presence of hyperoxia, however, was associated with a statistically significant increase in IL-1β expression when compared to control group (P = 0.05; Figure 4B). The administration of PJ in the presence of hyperoxia for both concentrations (PJ40 and PJ80) attenuated the surge in IL-1β (P = 0.03 and P = 0.02 respectively; Figure 4B). IL-6 expression demonstrated a significant increase after hyperoxia exposure when compared with controls. This increased expression of IL-6 was significantly attenuated in the hyperoxia + PJ80 but not in the hyperoxia + PJ40 as compared with the hyperoxia group (P = 0.03 and P = 0.5 respectively). Again, there was no difference in IL-6 expression between control and PJ groups (Figure 4C).

### TUNEL assay for apoptosis

**Figure 5:**
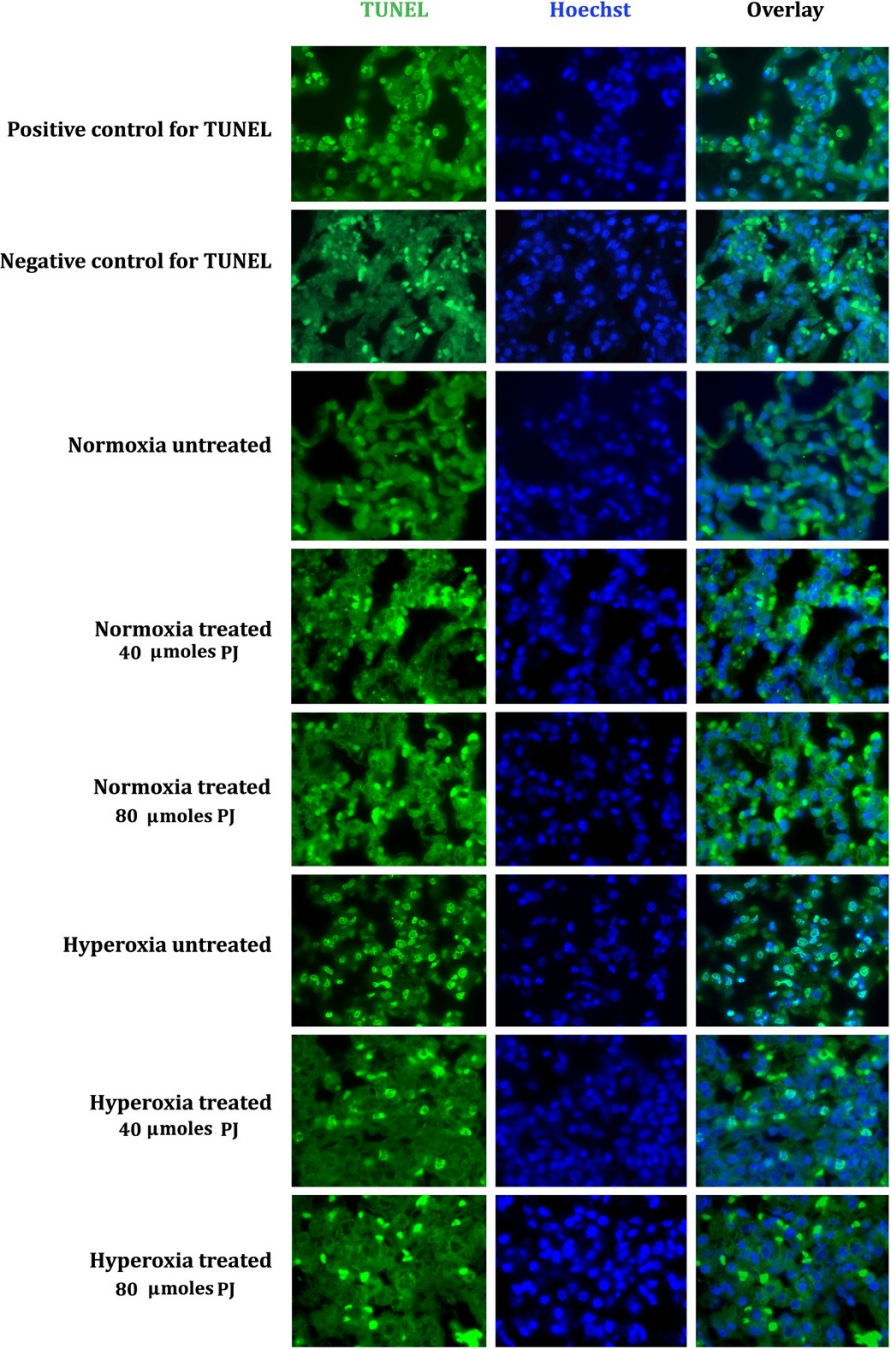
**Apoptosis assessment.** Terminal deoxynucleotidyl transferase- mediated dUTP nick-end labeling (TUNEL), Hoechst staining of lung sections and overlay of TUNEL and Hoechst for control, PJ40, PJ80, hyperoxia, hyperoxia + PJ40 and hyperoxia + PJ80.

No differences in apoptotic indices were observed between the control group and animals receiving PJ supplementation (40-μmol/kg and 80-μmol/kg). As noted before, the hyperoxia group did demonstrate an increase in apoptosis when compared with the control group [6]. Sections from hyperoxia + PJ groups revealed a significant reduction in apoptosis when compared to hyperoxia. The reduction in apoptotic activity was more prominent in the Hyperoxia + PJ80 group. Hoechst staining provided additional evidence that apoptosis did occur with hyperoxia exposure and did reverse in the Hyperoxia + PJ80 group as well (Figure 5).

### Pathological examination

**Figure 6:**
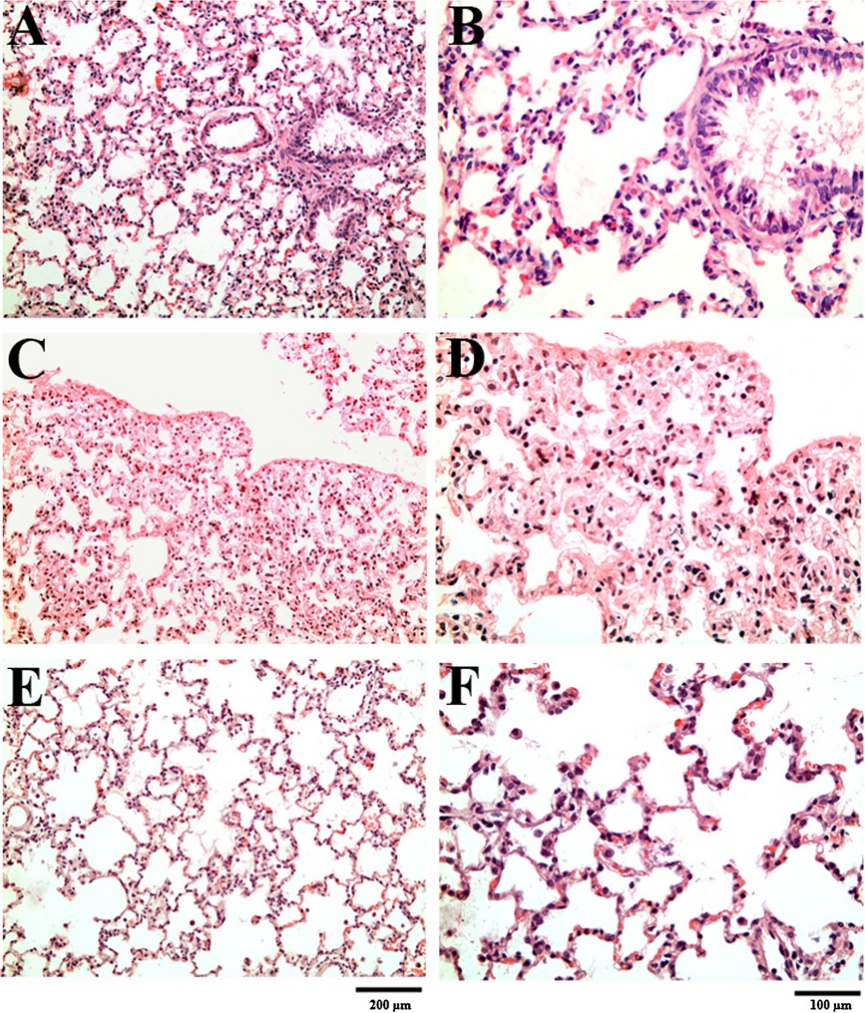
**Hematoxylin and eosin examination under light microscopy. A** &**B**, Normal rat lung showing thin interstitial alveolar wall and fine capillary vessels. Rare inflammatory cells in the wall and intra-alveolar spaces are noted and minimal swelling around blood vessels. **C** &**D** After five days of hyperoxia, thickening of the interstitial wall, capillary congestion and the inflammation is noted. At higher magnification, neutrophil and the increased mononuclear inflammatory cells within the wall and the alveolar spaces are observed. **E** &**F**: With PJ supplementation, a significant reduction in the thickness of the interstitial alveolar wall and the marked reduction in the number of inflammatory cells is noted. There is now resemblance to normal tissue in **A** &**B**.

There were no significant pathological differences on examination of the control and PJ groups. After 5 days of hyperoxia, macroscopic examination revealed edematous lungs with punctuate hemorrhage when compared with the control group. Examination with H & E showed that the hyperoxia group had significant interstitial and alveolar wall thickening accompanied by congestion of the capillary blood vessels and penetration of mononuclear inflammatory cells, mostly macrophages and neutrophils. Apoptotic bodies were also noted. By administering PJ, the damaging effects of hyperoxia were attenuated. There was a reduction interstitial wall edema and vascular congestion. No significant recruitment of inflammatory cells was noted and no apoptotic bodies were observed (Figure 6). Hyperoxia + PJ80 resulted in a protective effect against the pathologic changes associated with hyperoxia.

## Discussion

Our study did demonstrate the systemic effects of hyperoxia in an animal model. There was an increase in mortality in the hyperoxia only group and the remaining animals appeared ill and lost weight. The lungs were intensely affected as well. A significant increase in the W/D ratio and in albumin leak into the BALF was associated with hyperoxia. Hyperoxia was also associated with an increase in florescence of DHE, suggestive of an increased oxidative stress status of the lungs. This was coupled with a surge in the expressions of IL-1β and IL-6. Pathologically, alveolar and vascular congestion, increased infiltration of mononuclear inflammatory cells into lung parenchyma and increased apoptosis was also observed. Our findings are similar to those reported by others and us in the literature [21, 22]. In a time course animal study, Nagato et al. noted a significant increase in the number of macrophages and neutrophils after 24 hours of hyperoxia. Superoxide dismutase activity was decreased and glutathione/oxidized glutathione ratio was also reduced [23]. Previously, we have reported a surge of inflammatory mediators associated with hyperoxia that was also time dependent [6].

Pomegranate Juice supplementation reduced mortality as animals receiving daily 80-μmol/kg of PJ survived hyperoxia but continued to demonstrate significant weight loss suggesting persistent systemic effects of hyperoxia. In the lungs, PJ supplementation significantly reduced the increase in the W/D ratio, and albumin leak noted with hyperoxia. The increase in IL-1β and IL-6 noted with hyperoxia was significantly reduced as well. Pathologically, there was a reduction in the alveolar and vascular congestion and a significant reduction in the number of inflammatory cells recruited to the lung. The increase in oxidative stress noted with hyperoxia was reduced as well. We concluded that, in this animal model, supplementation of PJ to animals exposed to 5 days of hyperoxia significantly reduced the damaging effects of hyperoxia noted in the hyperoxia only group.

Our results are encouraging amidst the increased discontent and skepticism at the role of antioxidants in lung injury associated hyperoxia and at the role of antioxidants in ALI in general. The disappointing results with antioxidants are multifactorial and can be due to: a) poor choice of the specific antioxidant molecule, b) improper timing and c) pathophysiology that is complex and thus controlling one limb of the process may not alter the ultimate progression of lung injury. The design of this study played an essential role leading to the positive results. The timing and the dose of antioxidant proved critical. In our study, we initiated PJ supplementation two weeks prior to hyperoxia. At the time of insult with hyperoxia, animals were adequately primed with antioxidants leading to early and effective control of oxidative stress/imbalance created by hyperoxia and thus averting the snowballing effects of switching on important inflammatory cascades/mediators leading to ALI. In addition, we experimented with higher doses of PJ and noted unwanted systemic effects (animals appeared ill and lost weight) and lung injury was observed in controls receiving massive doses of PJ supplementation (320 μmoles and 1000 μmoles). Excessively higher doses of exogenous anti-oxidants may be associated with significantly low levels of ROS leading to immunosuppression and possible infection [13]. Too much of the good thing (i.e. antioxidants) can actually counterproductive and possibly toxic [24]. As for the choice of antioxidants, we utilized PJ as an antioxidant rather than utilizing a single molecule from the PJ mixture of antioxidants. Choosing one specific antioxidant and expecting it to resolve ALI as compared to the mix of antioxidants noted in PJ may not be as effective and may be the setup for failure. It appears that the different antioxidants constituents of PJ exert synergistic effect culminating in better control of oxidative stress [25]. Finally and due to the additional anti-inflammatory effects of PJ, we cannot rule out other pathways that were affected by PJ culminating in limiting the damaging effects of hyperoxic exposure in this animal model.

## Conclusion

Prolonged hyperoxia is documented to induce ALI, which is thought to be secondary to excessive ROS production. In this study using an animal model of hyperoxia induced ALI, PJ supplementation reduced ROS production, decreased inflammation, apoptosis, neutrophil infiltration of lung parenchyma and the surge in the expression of inflammatory mediators observed with hyperoxia.

## References

[1] Clark JM, Lambertson CJ (1971). Pulmonary oxygen toxicity. Pharmacol Rev.

[2] Asikainen TM, White CW (2004). Pulmonary antioxidant defenses in the preterm newborn with respiratory distress and bronchopulmonary dysplasia in evolution: implications for antioxidant therapy. Antioxid Redox Signal.

[3] Asikainen TM, White CW (2004). Pulmonary antioxidant defenses in the preterm newborn with respiratory distress and bronchopulmonary dysplasia in evolution: implications for antioxidant therapy. Antioxid Redox Signal.

[4] Boveris A, Chance B (1973). The mitochondrial generation of hydrogen peroxide. General properties and effect of hyperbaric oxygen. Biochem J.

[5] Gutowski M, Kowalczyk S (2013). A study of free radical chemistry: their role and pathophysiological significance. Acta Biochim Pol.

[6] Husari AW, Dbaibo GS, Bitar H, Khayat A, Panjarian S, Nasser M, Bitar FF, El-Sabban M, Zaatari G, Mroueh SM (2006). Apoptosis and the activity of ceramide, Bax and Bcl-2 in the lungs of neonatal rats exposed to limited and prolonged hyperoxia. Respir Res.

[7] Davies KJ (2000). Oxidative stress, antioxidant defenses, and damage removal, repair, and replacement systems. IUBMB Life.

[8] Saugstad OD (2003). Bronchopulmonary dysplasia-oxidative stress and antioxidants. Semin Neonatol.

[9] Perrone S, Tataranno ML, Buonocore G (2012). Oxidative stress and bronchopulmonary dysplasia. J Clin Neonatol.

[10] Chang LL, Subramaniam M, Yoder BA, Day BJ, Ellison MC, Sunday ME, Crapo JD (2003). A catalytic antioxidant attenuates alveolar structural remodeling in bronchopulmonary dysplasia. Am J Respir Crit Care Med.

[11] Perveen S, Patel H, Arif A, Younis S, Codipilly CN, Ahmed M (2012). Role of EC-SOD overexpression in preserving pulmonary angiogenesis inhibited by oxidative stress. PLoS One.

[12] Jung O, Marklund SL, Geiger H, Pedrazzini T, Busse R, Brandes RP (2003). Extracellular superoxide dismutase is a major determinant of nitric oxide bioavailability: in vivo and ex vivo evidence from ecSOD-deficient mice. Circ Res.

[13] Berkelhamer SK, Farrow KN (2014). Developmental regulation of antioxidant enzymes and their impact on neonatal lung disease. Antioxid Redox Signal.

[14] Tyson JE, Wright LL, Oh W, Kennedy KA, Mele L, Ehrenkranz RA, Stoll BJ, Lemon JA, Stevenson DK, Bauer CR, Korones SB, Fanaroff AA (1999). Vitamin A supplementation for extremely-low-birth-weight infants. National Institute of Child Health and Human Development Neonatal Research Network. N Engl J Med.

[15] Lee JW, Davis JM (2011). Future applications of antioxidants in premature infants. Curr Opin Pediatr.

[16] Faria A, Calhau C (2011). The bioactivity of pomegranate: impact on health and disease. Crit Rev Food Sci Nutr.

[17] Gil MI, Tomas-Barberan FA, Hess-Pierce B, Holcroft DM, Kader AA (2000). Antioxidant activity of pomegranate juice and its relationship with phenolic composition and processing. J Agric Food Chem.

[18] Seeram NP, Aviram M, Zhang Y, Henning SM, Feng L, Dreher M, Heber DJ (2008). Comparison of antioxidant potency of commonly consumed polyphenol-rich beverages in the United States. Agric Food Chem.

[19] Borges G, Crozier A (2012). HPLC-PDA-MS fingerprinting to assess the authenticity of pomegranate beverages. Food Chem.

[20] Cerdá B, Cerón JJ, Tomás-Barberán FA, Espín JC (2003). Repeated oral administration of high doses of the pomegranate ellagitannin punicalagin to rats for 37 days is not toxic. J Agric Food Chem.

[21] Nagato A, Silva FL, Silva AR, Bezerra FS, Oliveira ML, Belló-Klein A, Cristovao Porto L, Santos Valenca S (2009). Hyperoxia-induced lung injury is dose dependent in Wistar rats. Exp Lung Res.

[22] Ozdemir R, Yurttutan S, Talim B, Uysal B, Erdeve O, Oguz SS, Dilmen U (2012). Colchicine protects against hyperoxic lung injury in neonatal rats. Neonatology.

[23] Nagato AC, Bezerra FS, Lanzetti M, Lopes AA, Silva MA, Porto LC, Valença SS (2012). Time course of inflammation, oxidative stress and tissue damage induced by hyperoxia in mouse lungs. Int J Exp Pathol.

[24] Jain M, Chandel NS (2013). Rethinking antioxidants in the intensive care unit. Am J Respir Crit Care Med.

[25] Seeram NP, Adams LS, Henning SM, Niu Y, Zhang Y, Nair MG, Heber D (2005). In vitro antiproliferative, apoptotic and antioxidant activities of punicalagin, ellagic acid and a total pomegranate tannin extract are enhanced in combination with other polyphenols as found in pomegranate juice. J Nutr Biochem.

